# Incidence and Determinants of Acute Diarrhoea in Malaysia: A Population-based Study

**DOI:** 10.3329/jhpn.v29i2.7814

**Published:** 2011-04

**Authors:** K. Gurpreet, G.H. Tee, N.M. Amal, R. Paramesarvathy, C. Karuthan

**Affiliations:** ^1^Institute for Public Health, Ministry of Health, Jalan Bangsar, 50590 Kuala Lumpur, Malaysia; ^2^Institute for Medical Research, Ministry of Health, Jalan Pahang, 50588, Kuala Lumpur, Malaysia; ^3^Kuala Lumpur City Hall, DBKL Tower 1, Jalan Raja Laut, 50350, Kuala Lumpur, Malaysia; ^4^Department of Social and Preventive Medicine, University of Malaya, 50603, Kuala Lumpur, Malaysia

**Keywords:** Cross-sectional survey, Diarrhoea, Acute, Sociodemographic factors, Malaysia

## Abstract

Acute diarrhoea is a major health problem in many parts of the world, contributing to about 1.8 million deaths globally. The objectives of the study were to assess the incidence, determinants, and severity of acute diarrhoea in the population. A nation-wide cross-sectional survey involving about 57,000 respondents was conducted via face-to-face interview among eligible respondents of all ages. An acute diarrhoeal episode was defined as having three or more episodes of loose stools in any 24-hour period within the past four weeks before the interview. The severity was measured by duration of acute diarrhoea and associated symptoms. The variables tested as determinants were age, sex, ethnicity, the highest educational level, total monthly household income, and locality. Univariate, bivariate and multivariate procedures meant for complex study design were used in the analyses. The four-week incidence of acute diarrhoea was 5% [95% confidence interval (CI) 4.8-5.2]. The incidence of acute diarrhoea among the estimated population was the highest among young adults aged 20-29 years, Other Bumiputras (the pre-dominant ethnic group in East Malaysia), those with tertiary-level of education, those earning a monthly household income of less than RM 400, and rural dwellers. Only age, ethnicity, the highest level of education attained, and locality were significantly associated with acute diarrhoea in bivariate analysis. In multivariate analysis, these four variables were found to be the determinants of acute diarrhoea. Sex and monthly household income were excluded from the model. The mean duration of acute diarrhoea was 2.0 days (standard deviation 1.3). Forty-six percent of the respondents reported stomach cramps as an associated symptom. The findings revealed that acute diarrhoea is still a major public-health concern in Malaysia and grossly under-notified. There is a need for intensification of public-health intervention efforts to reduce the incidence of acute diarrhoea while improving surveillance and notification of the disease.

## INTRODUCTION

In many parts of the world, diarrhoea is still a major health problem (1-3). Globally, an estimated 1.8 million people die every year due to diarrhoeal diseases, of whom 90% are children aged less than five years, mostly in developing countries (4). In low and middle-income countries, diarrhoeal disease is one of the leading causes of the burden of disease (1). Even in developed countries, the social and economic impact of diarrhoeal diseases has been shown to be considerable (5, 6).

Several countries have conducted population-based studies on acute diarrhoea to estimate its magnitude and frequency (7-21). In Malaysia (Fig.), while data captured through the public-health surveillance and hospital surveillance systems exist, most studies concentrated on acute diarrhoea among children (22-25). Population-based studies measuring its burden and determinants in the general community are still lacking. Such information is fundamental in the planning and implementation of prevention and management strategies at the community level. In view of this, as part of a 10-yearly nationwide survey by the Ministry of Health on health and morbidity in Malaysia, acute diarrhoea was included as a research topic for the first time.

**Fig.**
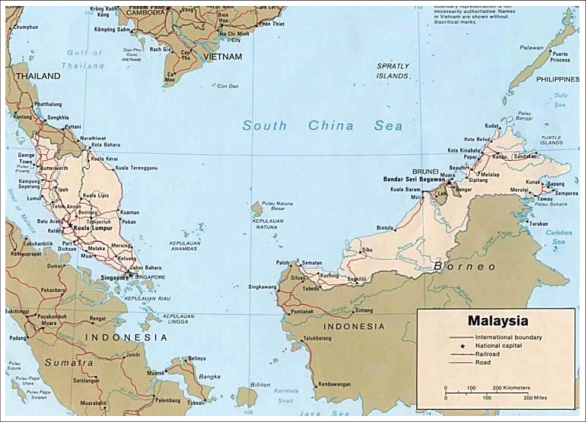
Ge ographical location of Malaysia

The general objective of the study was to determine the incidence of acute diarrhoea while the specific objectives were to determine the sociodemographic factors associated with acute diarrhoea, its predictors and to determine the severity of acute diarrhoea in the Malaysian population.

## MATERIALS AND METHODS

### Study design

This study was part of a nationwide cross-sectional community household interview survey, specifically known as the Third National Health and Morbidity Survey (NHMS III) (26). It was conducted throughout Malaysia from April to mid-August 2006. In total, 2,150 enumeration blocks (EBs) and 17,251 living quarters (LQs) were selected using a two-stage stratified random sampling method by the Department of Statistics Malaysia. All eligible persons in the household, who gave written consent, regardless of age, were interviewed. For children aged 12 years and below, the mother or guardian was interviewed on behalf of the child. The survey instrument for the acute diarrhoea module was a structured, pretested and piloted questionnaire administered via a face-to-face interview by trained interviewers comprising public-health nurses from the Ministry of Health and part-time recruits.

### Operational definition

Acute diarrhoea was defined as a self-reported episode of acute diarrhoea, i.e. having three or more episodes of loose stools in any 24-hour period within the past four weeks (28 days) before the interview. The definition excludes any type of chronic diarrhoea already experienced by the respondent as a result of the underlying diseases, such as cancer of the bowel, ulcerative colitis, or Crohn's disease.

Since the duration of an episode of acute diarrhoea is short, lasting usually a few days and rarely more than a week, an assumption for statistical analyses was made that each case of acute diarrhoea contributed to only one new episode of acute diarrhoea in the preceding four weeks of the interview. Since a person can acquire multiple episodes of acute diarrhoea in a year, the incidence of acute diarrhoea per person per year was also estimated (calculated from the estimated number of episodes annually divided by the total population for the year). Other assumptions made were that the incidence of acute diarrhoea and the population at risk were relatively stable throughout the year. The reference population was the 2006 projected population by the Department of Statistics Malaysia.

The severity of acute diarrhoea was measured by two parameters, namely the duration of diarrhoea as defined by the number of days the respondent experienced diarrhoea and the association of any other symptom(s), along with diarrhoea, such as fever, vomiting, stomach cramps, and blood in stools.

### Analysis of data

Appropriate quality-control measures were implemented at all levels of the survey from the field to the operations rooms to maximize response rate and completeness of data. Data were entered into a web-based database using double-entry method to minimize errors. Analyses of cleaned data were performed using the Complex Samples add-on module in the SPSS software (version 16) (27). For the purpose of analyses, non-respondents were those who had answered other sections of the questionnaire but not the module on acute diarrhoea. Respondents with a ‘do not know’ or missing answers were excluded from analyses.

### Ethical approval

The Medical Research Ethics Committee of the Ministry of Health approved the study.

## RESULTS

In total, 15,519 LQs were successfully visited giving an LQ sampling unit response rate of 90%. The response rate for this module from 56,710 eligible respondents representing 21,095,810 people on weighted counts was 98.3%.

The majority of the respondents comprised children and young adults aged 29 years and below (55.3%), females (52.9%), and Malays (57.4%). About 58% of the respondents were urban residents, and 62% had a household income of RM 1,999 or below. About 45% had attained a highest level of secondary education (Table 1).

**Table 1.** Frequency distribution of sociodemographic characteristics of respondents

Variable	No.	%
Respondents	55,748	98.3
Non-respondents	962	1.7
Age-group (years)		
0-4	5,912	10.6
5-9	6,629	11.9
10-19	10,900	19.6
20-29	7,497	13.5
30-39	7,136	12.8
40-49	7,325	13.2
50-59	4,810	8.6
60 and above	5,510	9.9
Unclassified	29	0.1
Gender		
Male	26,245	47.1
Female	29,503	52.9
Ethnic group		
Malay	31,986	57.4
Chinese	10,059	18.0
Indian	4,333	7.8
Other Bumiputras	6,954	12.5
Other	2,416	4.3
Educational level		
None	11,026	23.2
Primary	14,818	31.1
Secondary	18,008	37.9
Tertiary	3,344	7.0
Unclassified	372	0.8
Household income group (RM)*		
<400	4,617	8.3
400-699	8,447	15.2
700-999	6,454	11.6
1,000-1,999	14,992	26.9
2,000-2,999	8,419	15.1
3,000-3,999	4,215	7.6
4,000-4,999	2,018	3.6
≥5000	4,508	8.1
Unclassified	2,078	3.7
Locality		
Urban	32,212	57.8
Rural	23,536	42.2

*US$ 1=Approximately RM 3.04

### Incidence of acute diarrhoea

Overall, the incidence of self-reported acute diarrhoea within a four-week period in the Malaysian population was 5% [95% confidence interval (CI) 4.8-5.2] or 1,036, 518 episodes (95% CI 985, 935-1, 087, 100) in the weighted population. This translates to 13,474,728(95%CI12,875,828-14,099,955) episodes of acute diarrhoea annually. The distribution of incidence of acute diarrhoea by sociodemographic characteristics is shown in Table 2.

**Table 2.** Incidence of acute diarrhoea by sociodemographic characteristics and locality

Variable	Incidence						Crude OR		
	%	95 % CI		No. with sample	Estimated no. and population	p value	OR	95% CI	
		Lower	Upper						
Overall	5.0	4.8	5.2	2,799	1,036,518				
Age-group (years)						<0.001			
0-4	4.5	3.9	5.1	268	97,375		1.33	1.09-1.62	
5-9*	3.4	2.9	4.0	222	82,662		-	-	
10-19	6.3	5.8	6.8	685	253,840		1.92	1.62-2.28	
20-29	6.7	6.1	7.3	503	188,260		2.04	1.70-2.45	
30-39	5.2	4.7	5.8	369	138,440		1.56	1.30-1.87	
40-49	4.4	4.0	4.9	328	120,921		1.31	1.08-1.59	
50-59	3.9	3.4	4.5	219	80,945		1.16	0.93-1.43	
60 and above	4.1	3.6	4.8	203	73,328		1.22	0.99-1.52	
Gender						0.776	-	-	
Male	5.0	4.7	5.3	1,325	490,070		1.01	0.94-1.09	
Female*	5.0	4.7	5.3	1,474	546,447				
Ethnic group						<0.001			
Malay	5.4	5.1	5.8	1,729	641,911		1.55	1.36-1.76	
Chinese*	3.6	3.2	4.0	363	144,515		-	-	
Indian	5.1	4.3	5.9	221	87,564		1.43	1.18-1.75	
Other Bumiputras	5.8	5.2	6.6	406	135,062		1.67	1.40-1.98	
Others	3.2	2.5	4.0	80	27,463		0.89	0.68-1.16	
Educational Level						<0.001			
None*	4.1	3.7	4.5	445	164,109		-	-	
Primary	4.8	4.5	5.2	726	264,606		1.19	1.04-1.35	
Secondary	5.8	5.5	6.2	1,055	394,348		1.44	1.28-1.62	
Tertiary	6.4	5.5	7.3	214	83,851		1.58	1.33-1.89	
Household income-group (RM)** 0.758									
<400	5.5	4.8	6.3	249	87,966		1.07	0.87-1.33	
400-699	5.3	4.8	5.9	443	1,554,966		1.03	0.86-1.25	
700-999	4.7	4.1	5.3	302	108,802		0.91	0.74-1.10	
1,000-1,999	5.0	4.6	5.4	756	278,591		0.97	0.82-1.15	
2,000-2,999	5.0	4.5	5.6	426	161,353		0.97	0.80-1.17	
3,000-3,999	4.9	4.2	5.6	206	80,804		0.95	0.77-1.17	
4,000-4,999	4.9	3.9	6.1	97	38,596		0.95	0.72-1.26	
5,000 and above*	5.1	4.4	5.9	232	92,186		-	-	
Locality						0.020			
Urban*	4.7	4.5	5.0	1,518	621,701		-	-	
Rural	5.5	5.1	5.8	1,281	414,815		1.16	1.06-1.28	

*Reference group;

**US$ 1=Approximately RM 3.04

### Incidence of acute diarrhoea by sociodemographic profile

By age-group, the four-week incidence of acute diarrhoea was the highest among young adults aged 20-29 years (6.7%, 95% CI 6.1-7.3). By ethnicity, Other Bumiputras (the predominant ethnic group in East Malaysia) recorded the highest level of incidence (5.8%, 95% CI 5.2-6.6). By the highest educational level, those with tertiary education ranked the highest in the incidence of acute diarrhoea (6.4%, 95% CI 5.5-7.3). Those with less than RM 400 per month in household income showed the highest level of incidence (5.5%, 95% CI 4.8-6.3). Those residing in rural areas had a higher level of incidence of acute diarrhoea (5.5%, 95% CI 5.1-5.8) than urban dwellers. The males and females had the same level of incidence (5%, 95% CI 4.7-5.3).

### Bivariate analyses

Except for gender and household income, all other variables were significantly associated with acute diarrhoea (Table 2). Compared to respondents aged 5-9 years, respondents in all age-groups, except for those aged 50-59 and 60 years and above, were more likely to experience acute diarrhoea. The Malays, Indians, and Other Bumiputras were more likely to report acute diarrhoea than the Chinese. As the educational level increased from primary to tertiary, the likelihood of acute diarrhoea also increased steadily when compared with the reference group. Rural residents were 1.2 times more likely to report acute diarrhoea compared to urban residents.

### Multivariate analyses

Multivariate analyses were carried out to identify the determinants of acute diarrhoea. All the variables (age, gender, race, the highest educational level, household income, and locality) were initially included into the model. To obtain a parsimonious model, insignificant variables were removed one at a time, and only variables with a p value of <0.10 was retained. Only age, ethnicity, the highest educational level attained, and locality were the significant determinants in the final model (Table 3). Based on the adjusted odds ratio (AOR), those in the age-group of 10-19 years were 1.56 (95% CI 1.26-1.93) times more likely and those in the age-group of 20-29 years were 1.47 (95% CI 1.14-1.89) times more likely to experience acute diarrhoea compared to those in the age-group of 5-9 years. The Malays, Indians, and Other Bumiputras were 1.48 (95% CI 1.29-1.70), 1.41 (95% CI 1.15-1.71), and 1.62 (95% CI 1.36-1.94) times more likely to experience acute diarrhoea respectively compared to the Chinese. Compared to those with no formal education, the likelihood of acute diarrhoea among those with primary, secondary and tertiary education was 1.20 (95% CI 1.04-1.39), 1.39 (95% CI 1.20-1.61), and 1.63 (95% CI 1.33-2.00) respectively. Rural dwellers were only marginally (1.1 times) more likely to experience acute diarrhoea compared to urban dwellers (AOR=1.11, 95% CI 1.00-1.23).

**Table 3.** Final multivariate model for predictors of acute diarrhoea

Variable	Adjusted OR	95% CI	p value
Age-group (years)			0.000
0-4	1.44	1.11-1.87	
5-9*	-	-	
10-19	1.56	1.26-1.93	
20-29	1.47	1.14-1.89	
30-39	1.16	0.90-1.50	
40-49	1.00	0.78-1.28	
50-59	0.93	0.71-1.21	
60 above	1.09	0.84-1.41	
Ethnic group			0.000
Malay	1.48	1.29-1.70	
Chinese*	-	-	
Indian	1.41	1.15-1.71	
Other Bumiputras	1.62	1.36-1.94	
Others	0.83	0.63-1.10	
Educational level			0.000
None*	-	-	
Primary	1.20	1.04-1.39	
Secondary	1.39	1.20-1.61	
Tertiary	1.63	1.33-2.00	
Not applicable (0-4 years old)	0.89	0.683-1.17	
Locality			0.043
Urban*	-		
Rural	1.11	1.00-1.23	

*Reference group;

Gender and monthly household income were removed sequentially from the final model as these did not contribute significantly to the model (p>0.1).

CI=Confidence interval

OR=Odds ratio

### Duration of acute diarrhoea

The duration of acute diarrhoea in the population ranged from one day to seven days. The mean duration was 2.0 days (95% CI 1.9-2.1) (Table 4). More than one-third of the population experienced diarrhoea for only one day (42.2%; 95% CI 40.2-44.2) and two days (35.0%, 95% CI 33.1-36.9) respectively. Less than 4% experienced acute diarrhoea from four days to one week.

**Table 4.** Mean duration of acute diarrhoea by sociodemographic characteristics and location

Variable	Mean duration (days)	95% CI	p value
Overall	2.0	1.9-2.1	-
Age-group (years)			<0.001
0-4	2.7	2.4-2.9	
5-9	2.1	1.9-2.4	
10-19	2.0	1.9-2.1	
20-29	1.9	1.8-2.0	
30-39	1.8	1.7-2.0	
40-49	1.8	1.6-1.9	
50-59	2.1	1.9-2.2	
60 and above	2.0	1.8-2.1	
Gender			0.042
Male	2.0	1.9-2.0	
Female	2.1	2.0-2.1	
Etdnicity			<0.001
Malay	2.0	1.9-2.1	
Chinese	1.8	1.7-1.9	
Indian	2.1	2.0-2.3	
Otder Bumiputras	2.2	2.0-2.4	
Otders	2.5	2.1-2.8	
Educational level			<0.001
None	2.1	1.9-2.2	
Primary	2.0	1.9-2.1	
Secondary	1.9	1.8-2.0	
Tertiary	1.6	1.5-1.7	
Household income (RM)*			<0.001
<400	2.1	2.0-2.3	
400-699	2.1	2.0-2.3	
700-999	2.3	2.1-2.5	
1,000-1,999	2.0	1.9-2.0	
2,000-2,999	2.0	1.9-2.2	
3,000-3,999	1.8	1.6-1.9	
4,000-4,999	1.9	1.6-2.2	
5,000 and above	1.8	1.6-1.9	
Locality			0.036
Urban	2.0	1.9-2.0	
Rural	2.1	2.0-2.2	

*US$ 1=Approximately RM 3.04.

CI=Confidence interval

The longest mean duration of 2.7 days (95% CI 2.4-2.9) was observed among young children and infants. The mean duration of acute diarrhoea declined steadily among persons aged 5-49 years, with adults aged 40-49 years experiencing the shortest duration of 1.8 days (95% CI 1.6-1.9). From ages 50 years onward, the mean duration of acute diarrhoea was similar to that in older children and teens (Table 4). Women, other races, those without any formal education, those with a monthly household income of RM 700-999, and rural residents had the longest mean duration of acute diarrhoea (2.1-2.5 days; 95% CI 2.0-2.8).

### Associated symptoms

Abdominal cramp was the commonest symptom among those with acute diarrhoea. About 16% of the affected people complained of having vomiting or fever each (95% CI 14.6-17.5 and 14.4-17.5 respectively) while only 3.5% (95% CI 2.9-4.3) had dysentery. About 43% had acute diarrhoea without any other associated symptoms, such as fever, vomiting, or blood in stool.

## DISCUSSION

The study was the first nationwide population survey to obtain baseline estimates of acute diarrhoea in the community. The four-week incidence rate of acute diarrhoea of 5% in our study is comparable with the estimates by Scallan *et al.* in a study comparing Australia, Canada, Ireland, and the USA (16). The incidence was 7.6% in both Canada and USA, 6.4% in Australia, and 3.4% in Ireland. Although the response rates differed in that study, the use of a standard definition and the method of interview allow for comparison of the estimates among the four countries. Another study in Ireland found the incidence similar to our study at 4.5% (28). Hall *et al*. also found a similar estimate of 7% for infectious gastrointestinal illness among respondents in Australia (15). In contrast, other studies in the USA (10, 11) (July 1996–June 1997 and July 1998–June 1999), Canada (13, 14, 19), Queensland (29), and Cuba (8) found much higher estimates (8.6-15.5%) of acute diarrhoea compared to that in our study. In Malta, the rate for infectious intestinal disease was slightly lower than that of ours (3.2%) (21).

In our study, the highest incidence of acute diarrhoea was observed among young adults aged 20-29 years, followed by teenagers (10-19 years), and adults aged 30-39 years. Children aged less than five years had an incidence of acute diarrhoea that was slightly lower than in adults aged 30-39 years. A similar picture was observed in Queensland where the highest incidence was observed among persons aged 18-39 years, followed by children aged seven months to four years (29). Other studies have found the highest rates among children aged less than five years while the lowest rates were found among persons aged 65 years and above (10, 11, 16, 28). In our study, the lowest incidence rate was observed among children aged 5-9 years, followed by adults aged 50-59 years. A possible reason for the higher incidence of acute diarrhoea among young adults compared to other age-groups in our population could be attributed to their lifestyle and eating habits rather than their inherent susceptibility to develop intestinal infections. Being young healthy adults, they are likely to be single and more active and mobile compared to other age-groups. The lifestyle of these adults in the workforce or in higher institutions of learning puts them at a higher risk of exposure to food poisoning and gastroenteritis.

Although the prevalence of acute diarrhoea was higher among females in Norway (12), Canada (13, 14, 16, 17, 19), Australia (15, 16), Ireland (16), the USA (16), and the Netherlands (18, 20), no such finding was found in our study. Other studies in Argentina, the USA, Malta, and Australia also did not find any differences between the sexes (7, 10, 11, 21, 29). In Cuba however, males had higher morbidity due to acute gastrointestinal disease than females (8).

Our study found that the Chinese were least likely to report acute diarrhoea compared to other major ethnic groups. Many other studies have shown similar racial or cultural group differences in rates of acute diarrhoea (11, 14, 17). Although the reasons for such differences are not clear, the finding could be attributed to genetic or sociocultural differences between the races, which may be related to dietary and culinary practices or risk of acquiring gastrointestinal infections. The role of factors, such as genetics, immunity, and sociocultural component, in acquiring foodborne illnesses has been raised before (30, 31). Tam highlighted that, although little research has been done on behavioural, societal and cultural practices, these factors, including food choice and availability, consumer and societal attitudes towards food, and food-preparation practices, are part of the epidemiology of foodborne illness (30). In Malaysia, any Chinese cuisine typically is stir-fried, where the food is best served hot from the wok. Food that is thoroughly cooked and eaten immediately thereafter minimizes the risk of food poisoning.

Persons with a secondary or higher level of education were more likely to report acute diarrhoea than persons with no formal education. Similar findings were found in the USA and Queensland (10, 29). Other studies in the USA, Canada, and the Netherlands also showed that rates of gastrointestinal diseases increased as the level of education rose (11, 14, 18). Conversely, in Malta, the majority of cases had a lower level of education (21). Since social disadvantage, which is usually associated with lower levels of education, does not seem to be a risk factor in our study, it is likely that age may be a confounding factor. Young adults with the highest incidence of acute diarrhoea are more likely to have attained higher levels of education compared to older adults. Those with higher levels of education usually also have higher incomes, thus influencing their lifestyle, such as eating and travelling habits, and putting them at a greater risk of acquiring foodborne illnesses (18).

There was no difference in the incidence of acute diarrhoea among the different socioeconomic groups in our study. A similar finding was observed in other studies (10, 14, 29). In Australia, those with higher socioeconomic status had an increased risk of gastroenteritis (15). Rural residents had a higher incidence of acute diarrhoea compared to urban residents in this study. The finding is similar to that from a Canadian study (19). Elsewhere, studies have found either higher rates in urban rather than rural residents (10) or no difference in rates between urban and rural residents (11, 17, 29).

After adjusting for confounders, multivariate analyses revealed that only age, ethnicity, educational level, and locality were predictors of acute diarrhoea in the Malaysian population. In studies elsewhere, apart from age, income, gender, season, neighbourhood of residence, and health insurance were also the predictors for gastrointestinal illness (7, 8, 15, 17).

The severity of acute diarrhoea in the study was reflected by its mean duration and the presence of other associated symptoms experienced. The overall mean duration of acute diarrhoea in this study—which was two days—was comparable with that from Argentina, Cuba, and the Foodnet studies in the USA (7, 8, 10, 11). It must be cautioned that, in the US studies, acute diarrhoea was defined as self-reported diarrhoea that lasted for more than one day or was associated with impaired daily activity. Using a broader case definition, the average duration of illness in Canada was 3.7 days (14). A higher mean duration was also reported in Malta and Ireland (21, 28). While the range of duration for acute diarrhoea in our study was 1-7 days, in Argentina, the USA, and Canada, the range was much longer, lasting for at least four weeks (7, 10, 11, 14). The duration was comparable with findings from studies in Cuba and Ireland (8, 28).

Acute diarrhoea, in our study, was mostly associated with abdominal cramps followed by vomiting and fever. The findings concurred with those of the Foodnet study where more than half of the respondents with acute diarrhoea also had abdominal cramps (10, 11). In other studies, abdominal pain was the most common diarrhoea-associated symptom (8, 16). However, unlike our study where almost a similar proportion of the estimated population with acute diarrhoea had fever or vomiting, a higher proportion of respondents had fever rather than vomiting in Cuba and the USA (8, 10, 11). In Argentina, headache and muscle-pain were the most common secondary symptoms (7). Although there was a seasonal variation in the proportion of associated symptoms observed with acute diarrhoea in the Foodnet study, the trend was similar throughout the different seasons. The differences observed in the proportion of respondents with associated symptoms between the studies could be explained by the different definitions used. The definition for acute diarrhoea used in the Foodnet study implies that these cases were more severe compared to cases of acute diarrhoeal episodes only. Based on the duration and associated symptoms of acute diarrhoea in our study, we can conclude that >40.0% of the cases were mildly affected (no associated symptoms) and recovered quickly (within a day).

In Malaysia, it is a requirement by law to notify all new cases of cholera, typhoid, and paratyphoid, all forms of dysentery, and food poisoning. Diarrhoea is the common symptom of all these diseases. During 1990-2006, the number of annual notifications received for the above diseases ranged from 2,934 to 10,416 cases (32). In contrast, our study estimates at least 13 million episodes of acute diarrhoea annually. The figures indicate that acute diarrhoea in Malaysia is grossly under-reported, with only less than 0.1% of cases being captured by the national surveillance system annually. Under-reporting also occurs elsewhere (7, 33, 34). Estimates in England noted that only about one in six cases presents to the general practitioner, and of these, only a fraction was notified to the national surveillance system (34). This finding is not unexpected as the majority of the episodes are mild, not warranting medical treatments. Studies have shown that a sizeable proportion of acute diarrhoea can be attributed to food poisoning (35-38). In Malaysia, there is still a vast room for improvement in terms of the level of food hygiene and related practices among food-handlers and the general public. The implications for prevention of acute diarrhoea are significant.

### Limitations

The estimates from the present study have not been adjusted for the differences in the population structure. It has only been weighted to the 2006 projected population. Therefore, the actual burden of acute diarrhoea in the population, in terms of the total number of episodes per year, is likely to be more, assuming that the incidence rate throughout the year remains constant. In reality, seasonal, geographical and sociocultural factors are expected to influence local variations in the incidence of acute diarrhoea in the country throughout the year.

However, the strength of our study lies with its population-based study design, its high response rate, and minimization of recall bias, namely ‘telescoping’ using a short four-week recall period (34). One limitation of the study is the possibility of an individual experiencing two or more episodes of acute diarrhoea within the four-week recall period but reporting it as a single episode. The importance of using a validated definition for diarrhoea and episodes has been highlighted (39-41). It has also been suggested that three intervening diarrhoea-free days are optimum to define a new episode (39, 40). Our definition for duration of acute diarrhoea was non-specific and did not address any intervening diarrhoea-free days.

### Conclusions

This study was the first of its kind in Malaysia, providing baseline estimates for the incidence and distribution of acute diarrhoea among the general population. It highlights that acute diarrhoea is still a major public-health problem in Malaysia, with a four-week incidence of 5% in the estimated population. The 13,474,728 episodes of acute diarrhoea per year mostly affected teenagers and young adults. Since these young adults comprise the economically-productive population, efforts for the prevention and management of acute diarrhoea must be focused on this sub-population. Further research is recommended to study the reasons for the age, ethnicity, educational level, and locality differentials in the incidence of acute diarrhoea observed in this study. Understanding how social and cultural factors influence the incidence of acute diarrhoea is fundamental in planning long-term effective control measures.

National targets must be set to reduce the overall incidence of acute diarrhoea. A concerted effort to improve public-health surveillance and notification of the disease must also be made at the same time, if the effectiveness of intervention is to be evaluated. Intermittent population-based studies are recommended to estimate the true burden and compare trends of acute diarrhoea in the gene-ral population.

## ACKNOWLEDGEMENTS

Funding for this research was provided by the Ministry of Health, Malaysia. The authors thank the Director General of the Ministry of Health, Malaysia, for his permission to publish this paper. The authors also thank the members of the National Health and Morbidity Survey 2006 team for making this survey a success.
